# Graduate Study on the Continent—Germany

**Published:** 1908-08-22

**Authors:** 


### HOSPITAL ADMINISTRATION.

### CONSTRUCTION AND ECONOMICS.

### GRADUATE STUDY ON THE CONTINENT—GERMANY.

### IX.—MUNICH AND LEIPSIC.

The largest medical school in Germany is at Munich. Berlin, with its huge University, cannot rival the Bavarian capital in the number of its medical students, though it surpasses the latter in the total number of matriculates when all faculties are included. At Munich there are considerably more than a thousand medical students alone, and it may be thought that this fact is a striking proof of the recognised value and reputation of the Munich University as a school of medicine. This is, however, far from being the case.

#### Munich: Past and Present.

The excellence of a medical school does not depend on the mere number of students which it possesses, and there are many greater and far more important points for the post-graduate to consider than the numerical superiority of one University or centre over another. It must be borne in mind that many Continental centres, like some of our old London hospitals, live on their past reputation. When carefully compared with smaller and less well-known centres, these large and much frequented schools are found to be considerably inferior in value, so far, at least, as the post-graduate student is concerned, to their less well-advertised rivals. In the case of Munich this is easily seen. The University, so far as names go and reputation, is one of the best in Europe. One has only to glance down the list of the professors in the medical faculty, numbering such well-known men as Professors Grüber (hygiene), Von Voit (physiology), v. Bollinger (pathology), Muller (medicine), Kraepelin (psychiatry), Doderlein (gynæcology), Bezold (otology), Hoffmann (forensic medicine), Cremer (physiological chemistry), Lange (orthopædics), Gudden (anatomy), Pfaundler (pediatrics), Dieudonné (serum therapy), and von Bauer (medicine), to see that Munich need fear no comparison with any other Continental centre on the first point. As for the second, the part that the University has played in the advance of science, and in particular medical science, is by no means a small or insignificant one, while it is yearly turning out by far the largest number of practitioners who qualify from one special University centre in Germany. For these reasons Munich deserves some attention, and the post-graduate student, who will probably be advised on all sides to make it the Mecca of his "studien reise," will have carefully to consider its claims.

#### A Criticism and a Warning.

Without wishing for a moment to undervalue the usefulness of clinical study at Munich, we think it our duty to point out a few facts concerning post-graduate teaching at this centre, which the student should weigh before he makes up his mind to choose the Bavarian University in preference to any other centre. The amount of clinical material is great at Munich, but the number of students is also excessive, while the teaching staff, compared with that at Berlin or Leipsic, is small. Individual attention and individual opportunity are, therefore, not to be expected in the same degree as at these other centres. Again, while post-graduate courses are given at Munich, especially during the vacations, they cannot compare with those that are given at most other centres. No graduate should come to Munich unless he is amply provided with personal introductions. With such he will be in a privileged position and able to make the fullest use of the large amount of material which is undoubtedly available. Without these, as a mere free-lance, he will find attendance at the clinics hardly so profitable as at other, even much smaller centres.

#### Life in Munich.

Living is cheap and comfortable. There are many excellent pensions and hotels, and although as a general rule the graduate student who intends to stay for any length of time at a Continental centre should avoid both, at Munich there is much to be said in favour of pension life. The majority of pensions in the immediate neighbourhood of the clinics are beyond criticism—inexpensive, clean, comfortable, and quiet. As good are the private rooms, which can be hired at terms varying from 30-60 marks per month. Light and attendance are usually included in this price, but in every case the student should make a point of having a definite arrangement before he begins his occupation. Meals can be had at one or other of the numerous restaurants. They are usually cheap, and the food that is served is invariably good. The excellent beer for which the city is famous is too well known to need commendation here. According to the official students' calendar (issue 1908) rooms may be hired at from 20-35 marks per month, attendance 1-4 marks monthly, morning coffee 18-20 pfennigs, heating 20-30 pfennig, and lunch (Mittagtisch) from 50 pfennige. These figures are, perhaps, slightly under the average.

It would be impossible, within the limits of an article, to give anything like a full description of the many attractions of life in Munich. The city possesses an equable and agreeable healthy climate, though in winter the cold is sometimes excessive. There are fine parks (such as the magnificent Englische Garten), and the surrounding country offers opportunities for some of the most enjoyable excursions that the student, in search of romantic scenery, can wish to make. The artistic attractions of the city are as numerous and as inviting. The public galleries are well worth constant visiting, and the performances in the theatres and in the opera house rival those of any other German city. In fact, so various are these side attractions that it needs some self-denial and firm mindedness on the part of the student to attend regularly to his post-graduate work and not to play truant every second day.

#### Hospitals and Medical Courses.

Clinically the attractions are equally great. The hospitals, most of which have been rebuilt and improved while others are now being entirely reconstructed, are good examples of modern hospital construction, and the student will have many opportunities of availing himself of the rich material in them. The new Children's Hospital, the surgical clinics, and the large general hospital, are specially to be commended. We may add that in a short time Munich will possess the largest and best orthopædic hospital in Germany. A sum adequate for its construction and full equipment has been granted and building operations will be commenced before this article is published. This institution, which will rival the famous "Institutes" at Bologna and Milan, hitherto unsurpassed as orthopædic hospitals on the Continent, will be under the control of Professor Nange, whose work on the surgery of deformities has won him a world-wide reputation. The laboratories at Munich are up-to-date in every respect, and particularly in such branches as chemical physiology, metabolism, pathology, and bacteriology, the student will find this centre of special interest. With regard to attendance at ordinary lectures and courses, the university grants "guest tickets," available for one semester. Graduates will, however, find it more useful to matriculate and thus to share the privileges of full studentship. All that is necessary for matriculation is to produce proof of being a matriculate of some recognised university or of having passed an examination which is equivalent to what is demanded of the Bavarian student. In the case of diplomates, the diploma is usually sufficient, or at any rate, proof of having passed the usual preliminary examinations requisite for commencing medical study in England. The matriculation fee is 20 marks, and the usual students fees are exacted as in Berlin.

Post graduate courses at Munich are (*a*) Free, and (*b*) Private. The former are given under the auspices of the Central Committee for Post-graduate study in Germany, during both the summer and winter vacations. They are open to all qualified German practitioners, and, by special permission, to foreign graduates. The majority of them are monthly courses, both practical and theoretical, and comprising all subjects in medicine and its various branches. Like all the courses given under the auspices of this excellent association, they are exceptionally suited for graduates. Full particulars may be obtained on application to Prof. R. Kuttner, Kaiserin Friedrich Haus, Luisen Platz, Berlin, or to the local Secretary, Dr. A. Jordan, Munich. The private courses are usually limited to monthly courses in the autumn vacation, and are open to all qualified practitioners. A fee is charged in each case. Particulars may be obtained on application to Privat Docent Dr. Kerschensteiner, Ziemssenstrasse 1a, Munich.*

### Leipsic as a Medical Centre.

Like Munich, Leipsic is a large medical centre which is rapidly developing and bids fair, in a few years' time, to rival Berlin. Its clinical material is vast, its institutions are excellent, and its teaching staff includes such celebrities as Professors Trendelenburg (surgery), Hofmann (hygiene), Boehm (pharmacology), Flechsig (psychiatry), Curshmann (medicine), Marchand (pathology), Hering (physiology), Tillmans (topographical anatomy), Kölliker (orthopædics), Lane (pediatrics), and Kollmann (urology). The clinics are new and all are interesting, and the university, which is one of the oldest in Germany, enjoys a well-deserved reputation as a medical school. The matriculation-fee is 12m.; guest tickets, admitting to all lectures in one faculty for one term, are granted to foreign students on payment of 12m. and on production of the usual proofs of capacity. There is excellent opportunity for research work, the libraries being very complete and full. Living is slightly more expensive than in other large German towns, especially when pensions or hotels are chosen. Rooms average 30m. per month; during the winter term the prices are relatively higher. The medical student would do well to choose a lodging in the Südvorstadt, in the vicinity of the clinical laboratories. In the streets adjoining the University itself (Petersstrasse, Guinmaischstrasse, etc.) the prices are higher. Meals are to be obtained at any restaurant, but the prices are relatively higher than at Berlin or Munich.

### Graduate Courses at Leipsic.

Graduate courses are regularly given at Leipsic, both during the terms and in vacation time. Those in the vacations are to be recommended. They are free and private as at Munich, and application is to be made directly to the professor or demonstrator. Particular courses, with addresses of the demonstrators, may be obtained on application to Professor R. Kuttner, Kaiserin Friedrich Haus, Luisen Platz, Berlin. The following are a few of the courses available for graduates to attend:

University Ophthalmic Clinic: Three weeks' course in diseases of the eye.

University Medical Clinic: (Professor Dr. Curshmann): Course in physico-therapy.

Hygienic Institute: Course in practical bacteriology, three weeks.

University Gynæcological Polyclinic: Two weeks' course in gynæcological diagnosis on selected cases.

Children's Hospital: Three weeks' course in diseases of children.

Policlinic, Königstrasse 14 (Professor Kollmann): Three weeks' course in genito-urinary diseases; three weeks' course in practical cystoscopy; two weeks' course in practical urethroscopy.

University Otiatric Clinic (St. Jacob's Hospital): Three weeks' course in diseases of the ear; two weeks' course in operative otology.

Dermatological Clinic, Liebigstrasse: Three weeks' course in dermatology.

St. Jacob's Hospital, Polyclinic: Three weeks' course in surgical diagnosis (Professor Dr. Wilms).

The fees, it may be added, are exceptionally low.

Particulars of free courses are regularly published in the Journal of the Association for the Promotion of Graduate Study in Germany. Leipsic is by no means so well known to the foreign graduate as it deserves to be, and we feel confident that students who have once attended these excellent courses which are given here will desire to renew their acquaintance with the place on a future occasion.

### Research and Literature.

For research work there is plenty of material and opportunity at Munich. One advantage that this centre has over many others is that it is within easy reach of the large Italian, Austrian, and German centres, not to mention Paris and London, with which it is in daily connection by means of a fine express. The literary material at Munich itself is not, indeed, very rich, but for all practical purposes it is sufficient. We need not here stop to draw attention to the fact that for the graduate who wishes to "write up" the literature of the speciality which he is interested in, only the large centres such as London, Paris, and Berlin are really of use, for it is only at these large centres that he can find all the literary material at hand. Even at Vienna the libraries are incomplete and wanting in many necessities, and at Munich and Leipsic, however good they may be for purposes of study, for research purposes they are scarcely so well adapted.

* It may not be out of place here to add that inquiries regarding such courses generally, which may be made in English, French, or German, should be accompanied by full particulars regarding the applicant's status, the special subjects he is interested in, and, last but not least, by a properly addressed and stamped envelope for a reply. All such inquiries should be typed, or at least legibly written. Want of attention to these details adds considerably to the labours of the courteous general secretaries, who have to deal with a vast correspondence.

